# Identification and validation of mitochondrial and programmed cell death-related prognostic markers in pediatric acute myeloid leukemia

**DOI:** 10.3389/fimmu.2025.1671230

**Published:** 2025-11-20

**Authors:** Xiaoyan Hu, Qiang Zhao, Wei Deng, Yonghuan Li, Bei Liu

**Affiliations:** 1 The First Clinical Medical School, Lanzhou University, Lanzhou, China; 2 Department of Pediatric Hematology, Gansu Provincial Maternity and Child-care Hospital, Lanzhou, China; 3 Department of Hematology, The First Hospital of Lanzhou University, Lanzhou, China

**Keywords:** pediatric acute myeloid leukemia, programmed cell death, mitochondria, prognostic model, prognosis genes

## Abstract

**Background:**

Pediatric acute myeloid leukemia (AML) is characterized by poor prognosis and low survival rates following recurrence. While mitochondria and programmed cell death (PCD) are implicated in various diseases, their role in pediatric AML remains poorly understood. Identifying prognostic genes associated with PCD and mitochondrial function could enhance therapeutic approaches.

**Methods:**

Transcriptomic data and gene sets were sourced from public databases. Differentially expressed genes (DEGs) that intersected with PCD-related genes (PCD-RGs) and mitochondrial-related genes (mito-RGs) were selected as candidate genes. Regression analyses were performed to identify prognostic genes, which were then used to develop and validate a prognostic model. A nomogram was constructed, followed by functional analysis, immune microenvironment assessment, molecular regulatory network investigation, drug sensitivity profiling, and clinical validation through RT-qPCR.

**Results:**

Twenty-six candidate genes were identified, with three—PDHA1, OGG1, and OPA1—confirmed as potential prognostic markers through regression analyses. The prognostic model demonstrated robustness in both internal and external validations, and the nomogram exhibited good predictive power. Pathway enrichment analysis highlighted the involvement of DNA replication and epithelial-mesenchymal transition, alongside 14 differentially abundant immune cells (p < 0.05). Molecular network analysis indicated that hsa-miR-199a-5p regulates PDHA1 and OGG1. Drug sensitivity profiling identified potential therapeutic agents, including SB505124_1194. RT-qPCR validation confirmed consistent expression patterns for the prognostic genes.

**Conclusions:**

PDHA1, OGG1, and OPA1 were identified as potential prognostic markers for pediatric AML, providing valuable insights for the development of targeted therapeutic strategies. However, further validation in larger and more diverse clinical cohorts is still required to confirm its clinical applicability.

## 1 Introduction

Acute myeloid leukemia (AML) is a heterogeneous malignancy characterized by disrupted hematopoietic stem cell regulation and impaired differentiation, accounting for 15–20% of childhood leukemia cases (1, 2). Pediatric AML is associated with a poor prognosis, with a long-term survival rate of only 45–55% and a recurrence rate of approximately 30% (3, 4). The primary treatment strategies for AML include chemotherapy and hematopoietic stem cell transplantation (HSCT) (5). In pediatric AML, however, the failure rate of initial induction remission therapy is 10–15%, and only about one-third of patients with induction failure achieve eventual cure (6). This suboptimal prognosis is largely due to the inability to maintain disease remission after initial therapy. Chemotherapy causes significant toxicity, including myelosuppression, heightened infection risk, and long-term organ damage (7). Although allogeneic HSCT offers potential curative benefits, it carries risks such as graft-versus-host disease, opportunistic infections, and transplant-related mortality (8). Thus, identifying prognostic biomarkers and developing novel predictive models are crucial for the clinical management of pediatric AML. These efforts could yield new therapeutic targets, ultimately improving treatment outcomes.

Programmed cell death (PCD) is a regulated, sequential process that maintains a homeostatic balance between cell proliferation and cell death. This process encompasses 18 distinct forms, including apoptosis, necroptosis, and autophagy (9). Programmed cell death-1 (PD-1) expression has been observed in the leukemic cells of adult patients with AML, with reported frequencies ranging from 42% to 100%, but research on PCD in pediatric AML is limited (10). PCD is often regulated by intracellular organelles, particularly mitochondria, which play a pivotal role in inflammation associated with PCD. Dysregulation of mitochondrial dynamics can trigger various PCD pathways (11, 12). Notably, GSK621, an agonist of AMP-activated protein kinase (AMPK), has been shown to selectively eliminate AML cells, highlighting its potential as a therapeutic agent (13). However, the interaction between PCD and mitochondrial function in pediatric AML remains largely unexplored, hindering the development of targeted therapies for this population.

Utilizing extensive data resources on pediatric AML from public databases, this study applied a comprehensive suite of bioinformatics methods, including differential expression analysis, prognostic model construction, clinical correlation assessment, functional enrichment analysis, drug sensitivity prediction, and experimental validation. These approaches were used to conduct an in-depth investigation of prognostic genes associated with PCD and mitochondrial function in pediatric AML progression. The research also explored the molecular mechanisms implicated in pediatric AML and offered new insights into disease pathogenesis. This study provides a theoretical foundation for accurate prognostic diagnosis and targeted clinical treatment optimization for pediatric patients with AML.

## 2 Materials and methods

### 2.1 Data collection

The TARGET database was used to download the TARGET-AML cohort, which includes gene expression profiles, clinical data, and survival information from 187 pediatric AML tissue samples (accessed on 2023-11-20). Additionally, the GEO database was queried to obtain pediatric AML-related transcriptome datasets, including GSE2191 and GSE192638. GSE2191 (platform: GPL8300) contains tumor tissue data from 54 pediatric AML bone marrow samples and 4 control bone marrow samples, while GSE192638 (platform: GPL24676) served as an external validation set, comprising 41 pediatric AML bone marrow samples with survival data. To investigate PCD and mitochondrial functions in pediatric AML, 1,548 PCD-related genes (PCD-RGs) and 1,136 mitochondrial-related genes (mito-RGs) were sourced from relevant literature (9) (**Supplementary Table 1**) and the MitoCarta 3.0 database (accessed on 2023-11-20) (**Supplementary Table 2**).

### 2.2 Acquisition of candidate genes

Differential expression analysis was performed on the GSE2191 dataset to compare pediatric AML and control groups using the limma package (v 1.26.0) (14) (|log2FC| > 0.5, p < 0.05), identifying differentially expressed genes (DEGs). The results were visualized using ggplot2 (v 3.4.4) (15) for a volcano plot and Heatmap (v 4.1.0) (15) for a heatmap. The intersection of DEGs, PCD-RGs, and mito-RGs was used to identify candidate genes.

### 2.3 Enrichment analysis and construction of protein-protein interaction network

To further explore the biological functions and pathways associated with the candidate genes, GO and KEGG analyses were conducted using the clusterProfiler package (v 4.0.2) (16) (p < 0.05, count > 1). The top 10 biological functions from the GO analysis (p < 0.05) and the most significant pathways from the KEGG analysis (p < 0.05) were displayed. The candidate genes were input into the STRING database (with a confidence score threshold of > 0.4) to examine protein-level interactions, and the PPI network was visualized using Cytoscape software (v 3.10.1) (17).

### 2.4 Identification of prognostic genes

The 187 samples from the TARGET-AML cohort were randomly divided into two groups: 131 samples for the training set and 56 samples for the internal validation set, following a 7:3 ratio. The training set was used to identify candidate genes associated with pediatric AML prognosis. Univariate and multivariate Cox regression analyses were performed using the survival package (v 3.1-12) (18) to identify survival-associated genes (hazard ratio [HR] ≠ 1, p < 0.05), with the regression results tested for proportional hazards (PH) assumptions (p > 0.05). Forest plots were generated using the forestplot package (v 3.1.1) (19) to visualize the regression results, followed by further evaluation of the prognostic genes identified through these analyses.

### 2.5 Construction and validation of prognostic model

The risk score for pediatric AML was calculated using the following formula:

Risk score = h0(t) × exp(β1X1 + β2X2 + … + βnXn).

Subsequently, the surv_cutpoint function from the survminer package (v 0.4.6) (20) (based on the maximum selection rank statistic and log-rank test) was used to identify the optimal cutoff value within the queue, with minprop = 0.4 set to prevent extreme imbalance. Ultimately, pediatric AML patients were categorized into high-risk and low-risk groups. Next, survminer package (v 0.4.6) was utilized to draw risk curves and survival status plots to analyze the distribution of pediatric AML patients in different datasets as a whole. A heatmap illustrating the expression of prognostic genes between the two groups was created. Overall survival (OS) between the two groups was assessed using Kaplan-Meier (K-M) survival analysis with the survminer package (v 0.4.6). The diagnostic performance of the prognostic model was evaluated through receiver operating characteristic (ROC) curves at 1, 2, and 3 years using the survivalROC package (v 1.0.3) (21) (with area under the curve [AUC] ≥ 0.6). Using the same analytical approach, the model was further validated in both the internal and external validation sets.

### 2.6 Clinical correlation analysis between risk groups

To investigate survival differences between high-risk and low-risk groups based on clinical characteristics, common clinical factors in pediatric AML, such as age, CCAAT enhancer binding protein alpha (CEBPA) mutation, white blood cell (WBC) count (≥ 78.2757%, < 78.2757%), FAB classification (M0-M7), FLT3 ITD mutation, gender, and WT1 mutation, were included. Samples missing clinical data were excluded, and the remaining samples were categorized based on various clinical characteristics for correlation analysis. Stratified survival analysis for clinical factors was then performed across the two risk groups, with K-M curves plotted using the ggsurvplot function from the survminer package (v 1.0.3). The clinical characteristic grouping for training set samples is summarized in **Table 1**.

**Table 1:** Grouping of clinical characteristics in the training set samples.

N	107
Age	
≤9.214953	54
>9.214953	53
Gender	
Female	58
Male	49
CEBP mutation	
No	103
Yes	4
WBC_at_Diagnosis	
≥78.2757%	37
<78.2757%	70
FAB	
M0	3
M1	11
M2	29
M4	37
M5	23
M6	1
M7	3
FLT3 ITD mutation	
No	95
Yes	12
WT1 mutation	
No	101
Yes	6

### 2.7 Independent prognostic analysis

Next, using the survival package (v 3.1-12) and forestplot package (v 3.1.1), the risk score from the training set and the aforementioned clinical factors were combined for univariate and multivariate Cox regression analyses (p < 0.05) and PH assumption testing (p > 0.05). Independent prognostic factors were identified, and a nomogram was constructed using the rms package (v 6.1-0) (22). The nomogram model was then evaluated through calibration curves and ROC analysis for 1, 2, and 3 years.

### 2.8 Enrichment analysis based on risk score

In the training set, DEGs between high-risk and low-risk groups were identified using the DESeq2 package (v 1.38.0) (23). Log2 fold change (log2FC) was calculated, and genes were ranked from largest to smallest (p < 0.05, |log2FC| > 1). The clusterProfiler package (v 4.4.4) was used to perform Gene Set Enrichment Analysis (GSEA) with the “c2.cp.kegg_medicus.v2023.2.Hs.symbols.gmt” and “c5.go.v7.4.symbols.gmt” gene sets from MSigDB as the reference (p < 0.05, |normalized enrichment score (NES)| > 1). Gene set variation analysis (GSVA) was also conducted using the 50 hallmark gene sets from MSigDB, followed by differential analysis of GSVA scores between the two risk groups using the limma package (v 1.26.0). The low-risk group was used as the reference (t > 1 indicates activation of the pathway in the high-risk group, and t < -1 indicates activation in the low-risk group). The top 5 most significant functions from each enrichment analysis were visualized using the enrichplot package (v 3.19) (24).

### 2.9 Analysis of the immune microenvironment

This study employed the ssGSEA algorithm to infer immune cell infiltration from bulk RNA-seq data. Specifically, predefined gene sets for 28 immune cell types (25) were applied to log2-transformed expression matrices, generating enrichment scores for each immune cell type across the samples. The ssGSEA scores are dimensionless, rank-based enrichment values that reflect the relative abundance and activity of cell types, rather than absolute cell counts or proportions. To compare differences in immune cell infiltration levels between two risk groups, the Wilcoxon rank sum test (p < 0.05) was performed using the rstatix package (v 0.7.2) (26) and the ggplot2 package (v 3.4.4), identifying differentially immune cells. These immune cell distributions were visualized in box plots using ggplot2. To assess correlations among differential immune cells, correlation (cor) analysis was performed using the R package psych (v 2.2.9) (27) (|cor| > 0.3, p < 0.05), and results were visualized as heatmaps via the ggplot2 package (v 3.4.4). Subsequently, the quickcor function in the ggcor package (v 0.7.2) (28) was employed to analyze correlations between immune cells with differential infiltration and prognostic genes (p < 0.05), with correlation heatmaps generated using quickcor. Additionally, the ggdotchart function in the ggpubr package (v 0.6.0) (29) was utilized to create lollipop plots for visualization. Additionally, the rstatix and ggplot2 packages were used to compare the expression levels of 48 immune checkpoints (30) between the two risk groups, generating box plots (Wilcoxon rank-sum test, p < 0.05). The correlation between prognostic genes and differentially expressed immune checkpoints was then analyzed using quickcor (28) (|cor| > 0.3, p < 0.05).

### 2.10 Construction of molecular regulatory networks

The gene-gene interaction (GGI) network of prognostic genes was constructed *via* GeneMANIA. To further investigate the regulatory mechanisms of gene expression, miRNAs targeting prognostic genes were predicted using the miRwalk and starBase databases. The intersected miRNAs from both databases were then analyzed. Following this, lncRNAs regulating the intersected miRNAs were predicted using the starBase and miRNet databases, with the lncRNAs identified by both databases being intersected. The resulting lncRNA-miRNA-mRNA network was constructed to explore the regulatory relationships among prognostic genes.

### 2.11 Drug sensitivity analysis

To assess drug treatment response variability in pediatric AML, chemotherapy and targeted therapy drugs were sourced from the GDSC database. Using the oncoPredict package (v 0.5) (31), the half-maximal inhibitory concentration (IC50) for each patient’s response to chemotherapeutic and targeted therapy drugs was predicted. Correlations between drug IC50 values and risk scores (|cor| > 0.5, p < 0.05) were analyzed to infer drug sensitivity. Box plots were generated using ggplot2 to display significant differences in drug responses between the two risk groups (p < 0.05). Drugs showing the strongest positive and negative correlations with the risk score were presented. Additionally, small-molecule inhibitors were obtained from the Beat AML dataset (32), and drug sensitivity analysis was conducted similarly (|cor| > 0.4, p < 0.05).

### 2.12 Analysis of prognostic gene expression based on the GSE2191 dataset

Subsequently, to validate the expression differences of prognostic genes between the pediatric AML group and the control group, an analysis was conducted in the GSE2191 dataset, and the results were presented using box plots.

### 2.13 RT-qPCR

To further validate the expression of prognostic genes in clinical samples, five pairs of whole blood samples were collected from Gansu Provincial Maternity and Child-care Hospital, comprising five control samples (samples 1-5) and five AML samples (samples 6-10). The control donors were age-matched to the corresponding AML patients (± 2 years), with no known hematologic disorders, ensuring comparability between groups. This study was approved by the Institutional Review Board of Gansu Provincial Maternity and Child-care Hospital, with all participants providing informed consent prior to sample collection. Total RNA was extracted from approximately 50 mg of each tissue sample using TRIzol reagent (Ambion, Austin, USA) according to the manufacturer’s instructions. RNA concentration and purity were assessed using a NanoPhotometer N50, and samples with A260/A280 ratios between 1.8 and 2.0 were considered suitable for downstream applications. First-strand cDNA was synthesized from 2 µg of total RNA using the SureScript First-Strand cDNA Synthesis Kit (Servicebio, Wuhan, China) in a 20 µL reaction volume. The reverse transcription reaction was carried out under the following conditions: 25°C for 5 min, 50°C for 15 min, and 85°C for 5 sec, followed by hold at 4°C. RT-qPCR was performed using 2× Universal Blue SYBR Green qPCR Master Mix (Servicebio, Wuhan, China) on a CFX Connect Real-Time PCR System (Bio-Rad, USA). Each 10 µL reaction contained 3 µL of diluted cDNA, 5 µL of master mix, and 0.5 µM each of forward and reverse primers. The amplification protocol consisted of an initial denaturation at 95°C for 1 min, followed by 40 cycles of 95°C for 20 sec, 55°C for 20 sec, and 72°C for 30 sec. Melting curve analysis was performed to confirm primer specificity. The primer sequences were detailed in **Supplementary Table 3**. GAPDH was used as the endogenous control for normalization. Gene expression was quantified using the 2^-ΔΔCt^ method (33). Data visualization was conducted using GraphPad Prism 10 (34), with comparisons between groups assessed using the two-tailed Student’s t-test (unpaired). Statistical significance was set at p < 0.05.

### 2.14 Statistical analysis

All statistical analyses were conducted using R software (version 4.2.2; R Foundation for Statistical Computing, Vienna, Austria). Specifically, the clusterProfiler package was used for GO and KEGG enrichment analysis, the limma package for gene differential expression analysis, the rms package for plotting nomogram, and calibration curves, and the survivalROC package for ROC analysis. Differences between groups were compared using the Wilcoxon rank-sum test (p < 0.05). Survival analysis was conducted with the log-rank test to evaluate group differences (p < 0.05).

## 3 Results

### 3.1 Identification of candidate genes, enrichment analysis, and PPI construction

**Figure 1:**
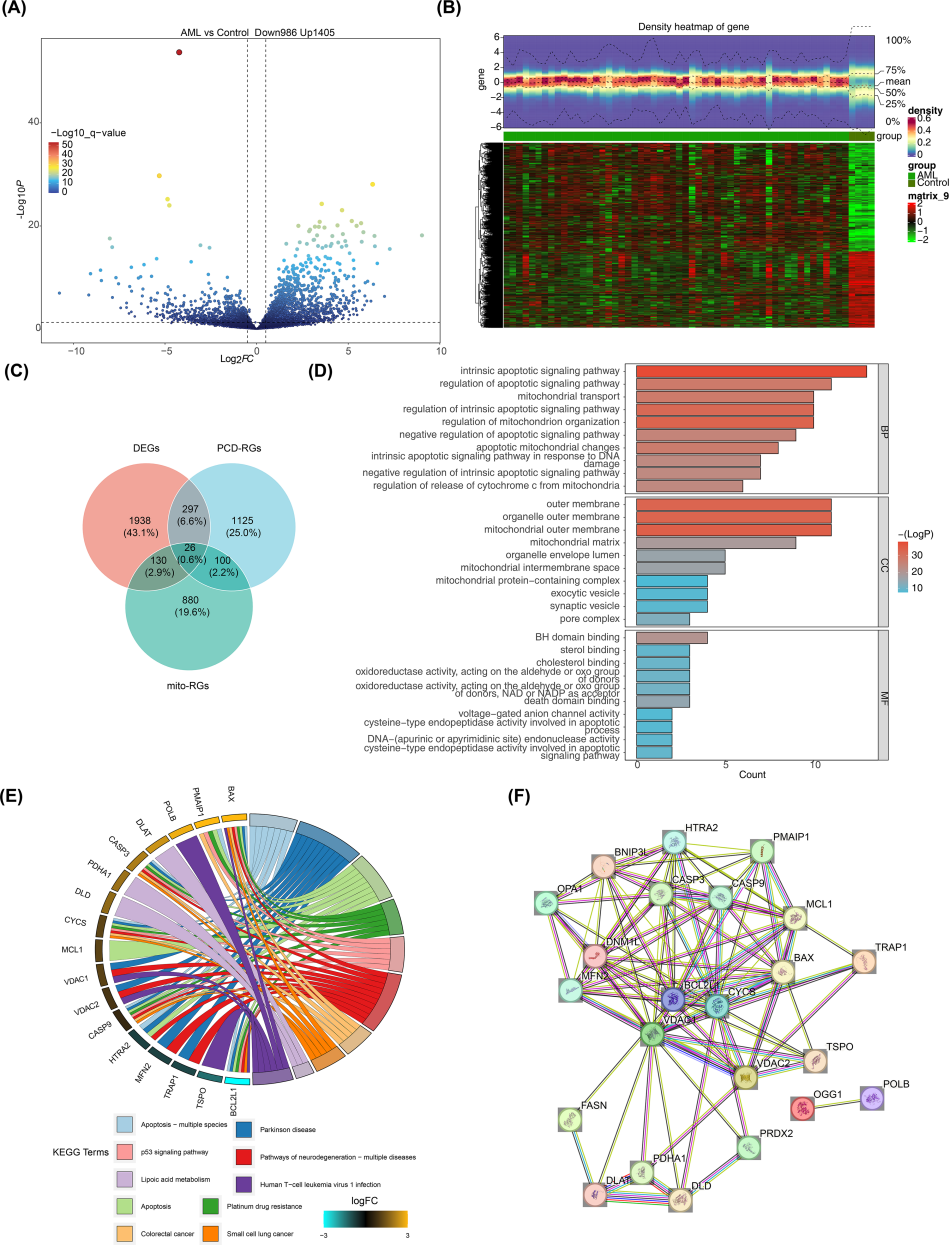
Screening for DEGs. **(A)** Volcano plot of DEGs in GSE2191. Differential expression was assessed with limma using |log2FC| > 0.5 and p < 0.05; genes with log2FC > 0.5 (up) or < -0.5 (down) are highlighted. **(B)** Heatmap of DEGs in GSE2191. Expression values were normalized (z-score by gene) and clustered using Euclidean distance and complete linkage. **(C)** Venn diagram showing intersection of DEGs, PCD-related genes (PCD-RGs), and mitochondrial-related genes (mito-RGs) to define candidate genes. **(D, E)** Functional enrichment analysis of candidate genes via Gene Ontology (GO: BP, CC, MF) and KEGG pathways performed with clusterProfiler (over-representation analysis; p < 0.05, count > 1). Top terms/pathways are shown. Multiple testing correction: Benjamini–Hochberg (BH) where applicable; terms reported meet p < 0.05 after correction or as indicated in the main text. **(F)** Protein–protein interaction (PPI) network of candidate genes obtained from STRING (confidence score > 0.4) and visualized in Cytoscape.

Differential expression analysis of the GSE2191 dataset revealed 2,391 DEGs, with 1,405 upregulated and 986 downregulated genes in the pediatric AML group (**Figure 1A**). A heatmap generated using these DEGs successfully distinguished the pediatric AML and control groups (**Figure 1B**). The intersection of DEGs, PCD-RGs, and mito-RGs identified 26 candidate genes (**Figure 1C**; **Supplementary Table 4**). Subsequently, enrichment analysis was performed to understand the functions and related pathways of the candidate gene. GO enrichment analysis revealed a total of 441 enriched terms, including 365 biological processes (BPs), 21 cellular components (CCs), and 55 molecular functions (MFs) (**Supplementary Table 5**). The top 10 gene functions identified included intrinsic apoptotic signaling pathway, mitochondrial outer membrane, and BH domain binding (**Figure 1D**). KEGG enrichment analysis identified 52 pathways (**Supplementary Table 6**), with the top 10 significantly enriched pathways including apoptosis, platinum drug resistance, and p53 signaling (**Figure 1E**). PPI networks are essential for understanding the structure and function of cellular networks, as well as the pathogenesis of diseases. To explore potential interactions among the 26 candidate genes, a PPI network was constructed, which included 23 nodes and 96 edges. Key genes such as OPA1, PDHA1, and BCL2L1 were identified in the network (**Figure 1F**).

### 3.2 Construction and validation of prognostic model based on prognostic genes

**Figure 2:**
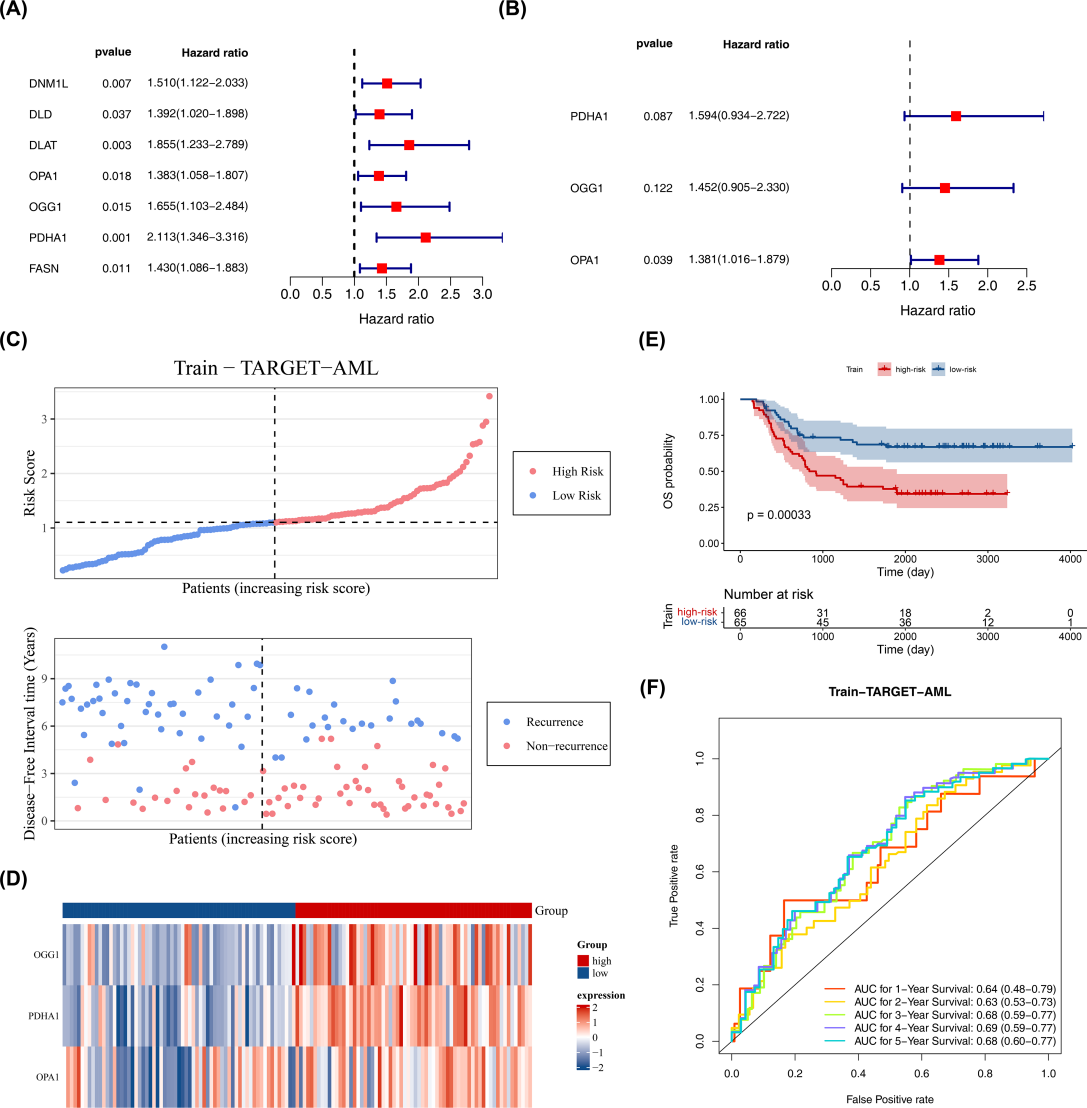
Construction of prognostic model. **(A)** Univariate Cox proportional hazards regression of candidate prognostic genes in the training set (n = 131). Hazard ratios (HR) with 95% confidence intervals (CI) and p values are shown; genes with p < 0.05 were considered significant. Proportional hazards (PH) assumption was tested (p > 0.05 indicates PH satisfied). **(B)** Multivariate Cox regression of selected prognostic genes; HR (95% CI) and p values shown; PH assumption tested (p > 0.05). **(C–E)** Risk curve, survival status plot, heatmap of prognostic gene expression, and Kaplan–Meier K-M survival analysis comparing high- and low-risk groups defined by surv_cutpoint (maximally selected rank statistic; minprop = 0.4). K-M curves were compared with the log-rank test; p values are reported. **(F)** Diagnostic value of the prognostic model in GSE2191 assessed by time-dependent ROC curves (survivalROC) at 1, 2, and 3 years; area under the curve (AUC) is reported. Statistical methods: Cox regression (survival package), log-rank test for K-M, ROC AUC with confidence intervals.

Based on 26 candidate genes, this study further explored which genes hold significant prognostic value for survival outcomes in pediatric AML. Using 131 samples from TARGET-AML (the data was randomly split into a training set of 131 cases and a test set of 56 cases in a 7:3 ratio), through univariate Cox regression analysis, 7 candidate prognostic genes were identified, among which these genes were all considered as high risk genes (HR > 1) (**Figure 2A**). Multivariate Cox regression analysis and the PH assumption test (p > 0.05) further confirmed 3 potential prognostic genes—PDHA1, OGG1, and OPA1—as significant (**Figure 2B**; **Table 2**).

**Table 2:** PH assumption test.

Gene	chisq	df	P
PDHA1	2.363675	1	0.124189
OGG1	0.36684	1	0.544732
OPA1	1.475837	1	0.224427
GLOBAL	13.62714	7	0.058225

The prognostic model was constructed as follows: Risk score = h0(t) × (PDHA1 × 0.46653 + OGG1 × 0.373 + OPA1 × 0.32316). Using the optimal cutoff value (cutpoint = 1.09), the 131 pediatric patients with AML were divided into high-risk (66 samples) and low-risk (65 samples) groups (**Figure 2C**). The survival status plot indicated that higher risk scores correlated with a greater number of deceased patients (**Figure 2C**). A heatmap based on the prognostic genes effectively distinguished the two groups (**Figure 2D**). K-M survival curves showed that the high-risk group had significantly lower survival rates (p < 0.05) (**Figure 2E**), with AUCs for 1, 2, and 3 years all greater than 0.6, demonstrating that the prognostic model effectively predicts the survival of pediatric patients with AML (**Figure 2F**).

**Figure 3:**
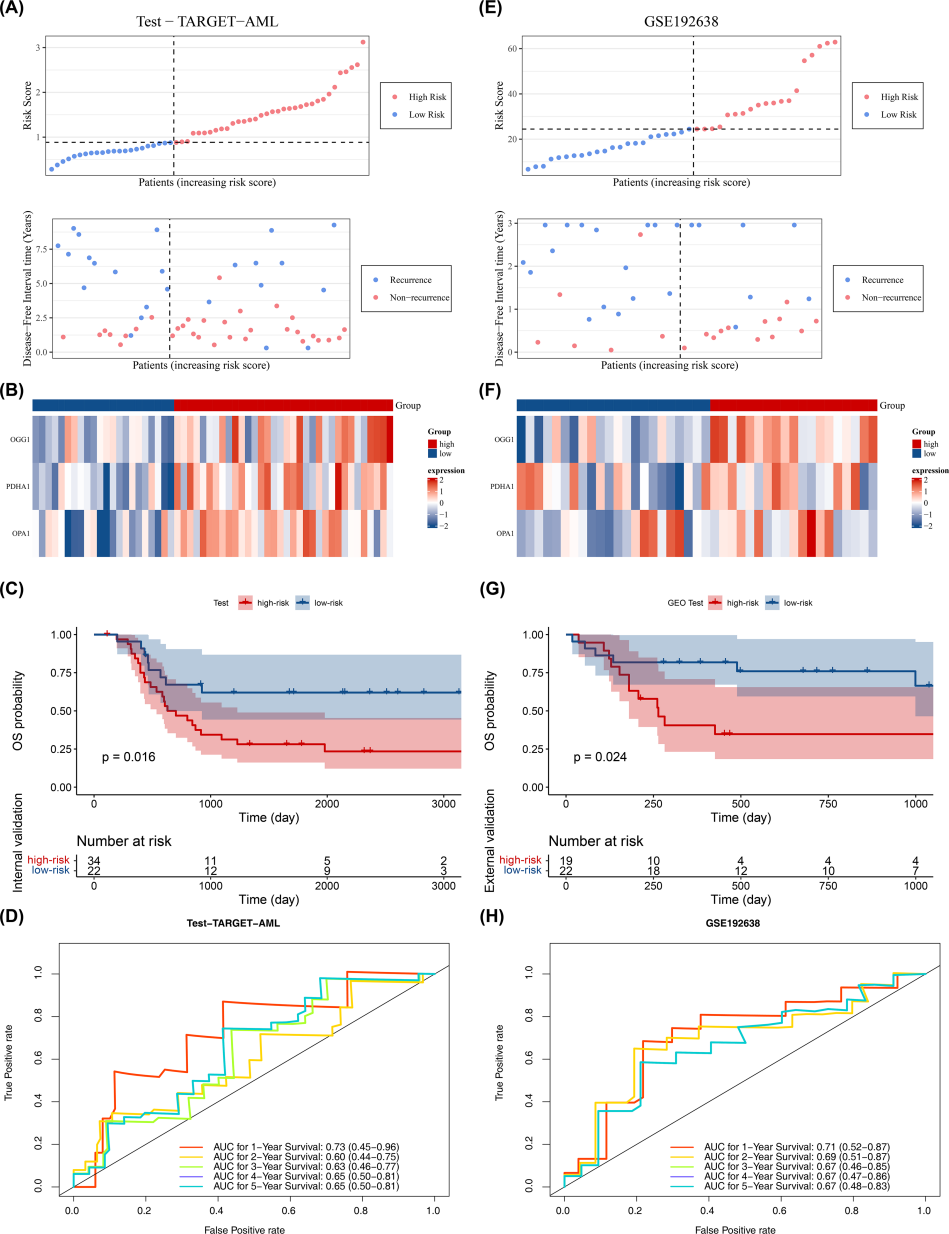
Validation of prognostic model. **(A–D)** Internal validation set (n = 56): survival status plot, heatmap, Kaplan–Meier survival analysis (log-rank test), and ROC curve (time-dependent ROC; AUC reported). **(E–H)** External validation set (GSE192638, n = 41): survival status plot, heatmap, Kaplan–Meier survival analysis (log-rank test), and ROC curve (time-dependent ROC; AUC reported). Statistical tests and thresholds are the same as **Figure 2**. K-M p values from log-rank test; ROC AUC with 95% CI.

The model’s reliability was confirmed through internal (from TARGET-AML) and external validation (GSE192638). In the internal validation set, the prognostic model identified an optimal threshold (0.876), dividing the cohort into high-risk (34 samples) and low-risk (22 samples) groups. The survival status plot, heatmap, and K-M curve results (p < 0.05) were consistent with the training set findings (**Figures 3A–C**). ROC analysis showed AUCs greater than or equal to 0.60 for 1, 2, and 3 years (**Figure 3D**). In the external validation set, the prognostic model identified an optimal threshold (24.43), dividing the cohort into high-risk (19 samples) and low-risk (22 samples) groups. The survival status plot, heatmap, K-M curve (p < 0.05), and ROC curve results were consistent with the internal validation set (**Figures 3E–H**). These results confirm the robustness of the prognostic model in assessing the risk of pediatric patients with AML.

### 3.3 Stratified survival analysis

**Figure 4:**
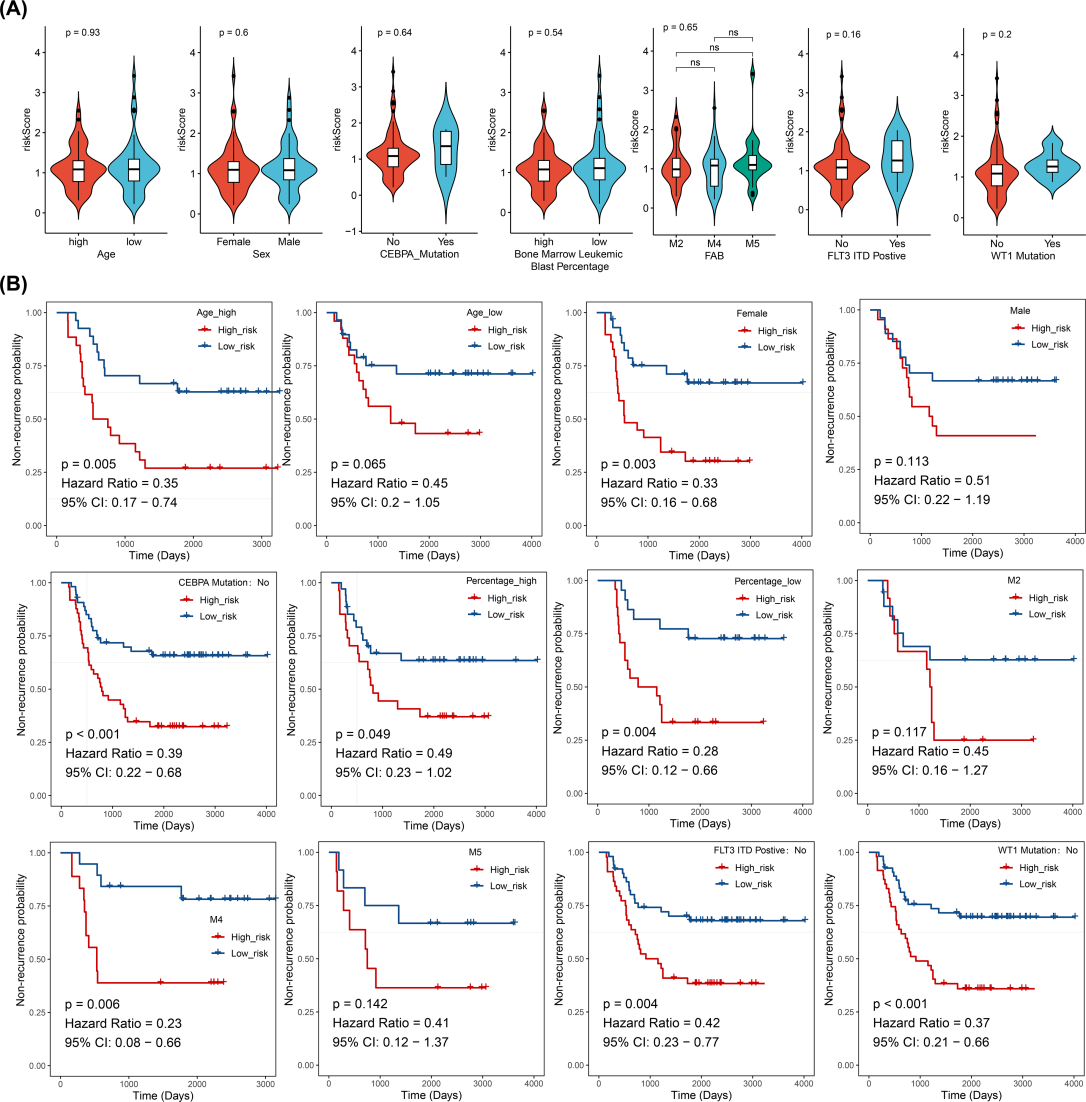
Stratified survival analysis between risk models and clinical characteristics (log-rank test), (ns, not significant). **(A)** Distribution of risk scores across different clinical pathological subgroups. **(B)** Kaplan-Meier curves for high- and low-risk groups within different clinical pathological subgroups.

In the training cohort (from TARGET-AML), stratified analysis based on clinical characteristics revealed significant differences in DFS status between the two risk groups in several subgroups, including those with high age, female gender, CEBPA wild-type, elevated WBC count, FAB classification M4 stage, FLT3-ITD wild-type (No), and WT1 wild-type (No) (p < 0.05). KM curves for each subgroup demonstrated that patients with higher risk scores had poorer prognosis (**Figure 4**).

### 3.4 Construction and evaluation of pediatric AML prediction model

**Figure 5:**
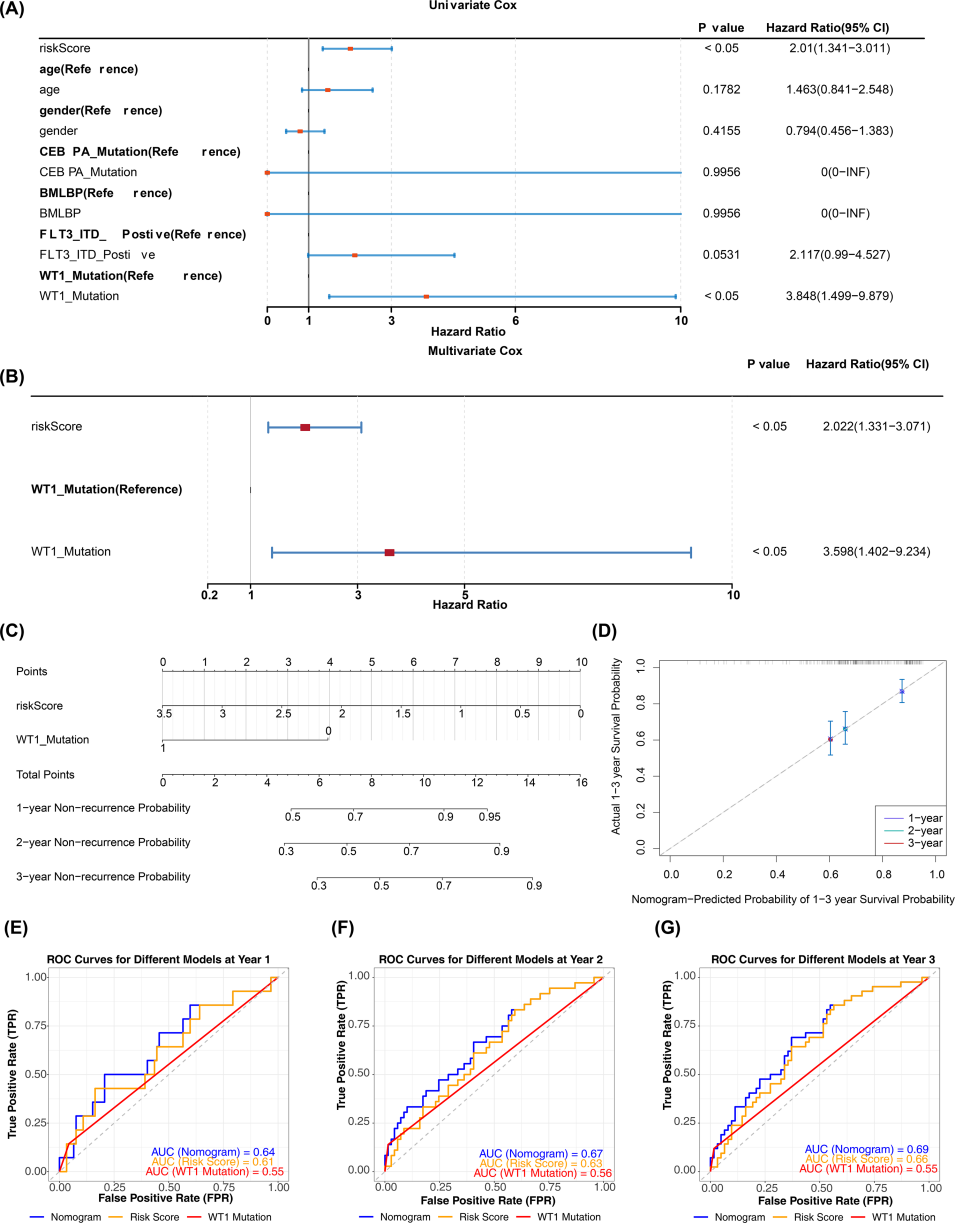
Construction and validation of nomogram. **(A, B)** Univariate and multivariate Cox regression analyses combining clinical variables and risk score (HR, 95% CI, p values). Variables with p < 0.05 in multivariate analysis were considered independent prognostic factors. PH assumption tested for each model (p > 0.05 indicates satisfied). **(C)** Nomogram incorporating independent prognostic factors (risk score and WT1 mutation) to predict 1-, 2-, and 3-year overall survival. **(D)** Calibration curves comparing predicted vs observed survival at 1, 2, and 3 years; calibration assessed by 1,000 bootstrap resamples. **(E–G)** ROC curves for independent prognostic factors and the nomogram at 1, 2, and 3 years (time-dependent ROC); AUC values and 95% CIs are reported.

In the TARGET-AML training cohort, after incorporating the aforementioned common clinical characteristics and risk scores, regression analyses and PH assumption tests identified risk score (p < 0.05, HR = 2.022, 95% CI = 1.331–3.071) and WT1 mutation (p < 0.05, HR = 3.598, 95% CI = 1.402–9.234) as independent prognostic factors (**Figures 5A, B**). The nomogram indicated that risk score had the most significant impact on patient survival, followed by WT1 mutation. A higher total score correlated with a higher probability of non-relapse, though the likelihood of non-relapse progressively decreased with extended timeframes (1, 2, and 3 years) at the same total score (**Figure 5C**). The calibration curve confirmed that the survival probabilities for different years closely matched the reference line (**Figure 5D**). Diagnostic evaluation revealed that the AUC values of the nomogram model surpassed those of individual prognostic factors (AUCs > 0.6), demonstrating its strong predictive performance (**Figures 5E–G**).

### 3.5 GSEA and hallmark pathway differential analysis

**Figure 6:**
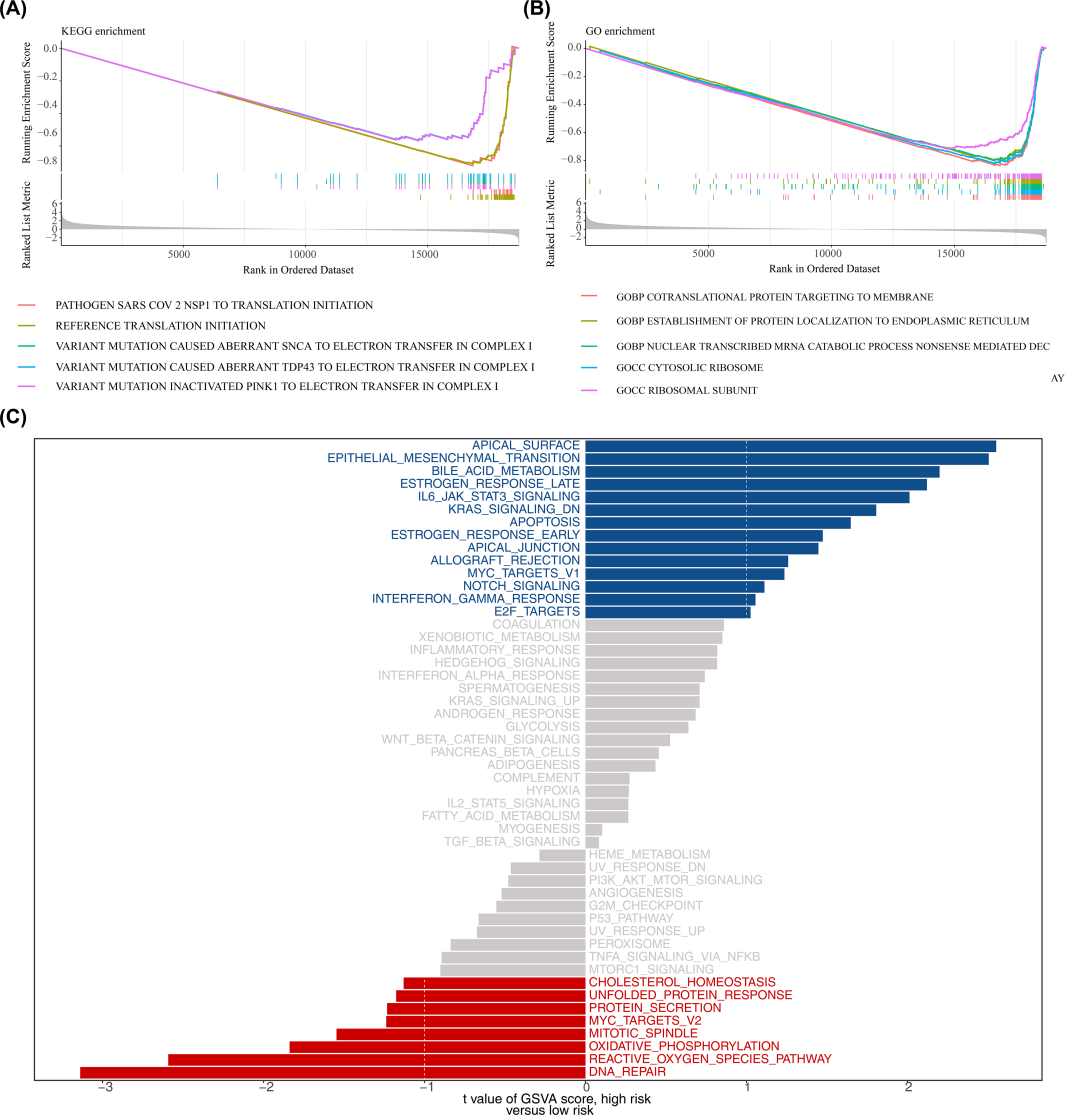
Functional enrichment analysis. **(A, B)** Gene Set Enrichment Analysis (GSEA) for KEGG and GO gene sets (MSigDB c2.cp.kegg and c5.go; permutations = gene_set; p < 0.05 and |normalized enrichment score (NES)| > 1 considered significant). Adjusted p values (BH) are reported where applicable. **(C)** Gene Set Variation Analysis (GSVA) comparing high- vs low-risk groups using the 50 Hallmark gene sets; differential GSVA scores were tested using limma (empirical Bayes moderated t-statistic); pathways with adjusted p < 0.05 (BH) and |t| > 1 are shown.

In the high- and low-risk groups of the training set, based on the KEGG gene set (c2.cp.kegg_medicus.v2023.2.Hs.symbols.gmt) from GSEA analysis, some pathways, such as reference translation initiation, were then observed (**Figure 6A**; **Supplementary Table 7**). GO gene set (c5.go.v7.4.symbols.gmt) analysis identified processes like cotranslational protein targeting to the membrane (**Figure 6B**; **Supplementary Table 8**). Additionally, GSVA analysis indicated that apical surface and epithelial-mesenchymal transition (EMT) pathways were activated in the high-risk group, while DNA repair pathways were activated in the low-risk group (**Figure 6C**; **Supplementary Table 9**). The findings of this study suggested that DNA repair and epithelial-mesenchymal transition may play a role in specific risk groups; however, given the small sample size and potential cross-platform bias in this study, the reliability of this conclusion requires validation in future studies with larger sample sizes.

### 3.6 Description of the immune microenvironment in pediatric AML

**Figure 7:**
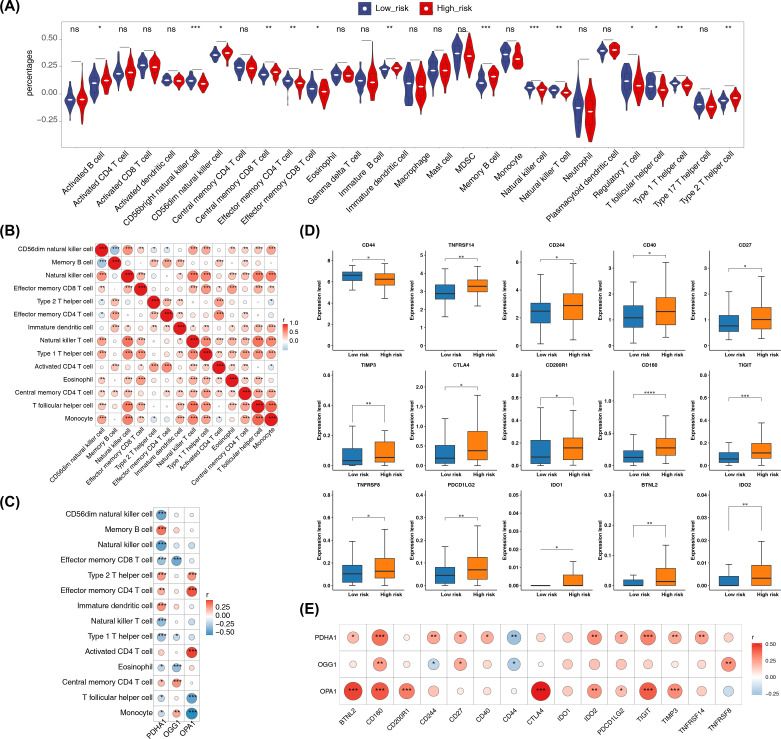
Immune microenvironment analysis. **(A)** Differences in immune cell infiltration (ssGSEA scores for 28 immune cell types) between high- and low-risk groups. ssGSEA was applied to log2-transformed expression matrix; group comparisons used Wilcoxon rank-sum test (two-sided); p < 0.05 considered significant. **(B)** Correlation heatmap of differentially abundant immune cells computed by Spearman correlation (or Pearson as specified in Methods); correlations with |cor| > 0.3 and p < 0.05 are highlighted. **(C)** Correlation heatmap between prognostic genes and differential immune cells using Spearman correlation (p < 0.05); |cor| > 0.3 indicated on heatmap. **(D)** Immune checkpoint expression comparisons (48 checkpoints) between risk groups using Wilcoxon rank-sum test (two-sided); p < 0.05 considered significant. **(E)** Correlation heatmap between prognostic genes and differentially expressed immune checkpoints by Spearman correlation (|cor| > 0.3, p < 0.05). Significance annotations throughout: ns (p > 0.05); *p < 0.05; **p < 0.01; ***p < 0.001; ****p < 0.0001.

In addition, there were 14 immune cells with significant differences in infiltration levels between the high- and low-risk groups in the training set, including memory B cells, CD56dim natural killer cells, and natural killer cells (p < 0.001) (**Figure 7A**). Correlation analysis revealed that more than half of the differential immune cells had positive correlations, with the strongest positive correlation between monocytes and follicular helper T cells (cor = 0.757, p < 0.001) (**Figure 7B**; **Supplementary Table 10**). Additionally, OPA1 showed the strongest positive correlation with activated CD4 T cells (cor = 0.495, p < 0.001) (**Figure 7C**; **Supplementary Table 11**). In the high-risk group, 15 immune checkpoints were significantly expressed, including TNFRSF14 (p < 0.01) (**Figure 7D**). The strongest positive correlation was found between CTLA4 and OPA1 (cor = 0.513, p < 0.001), while CD44 and PDHA1 exhibited the strongest negative correlation (cor = -0.269, p < 0.001) (**Figure 7E**; **Supplementary Table 12**).

### 3.7 Molecular regulatory networks

**Figure 8:**
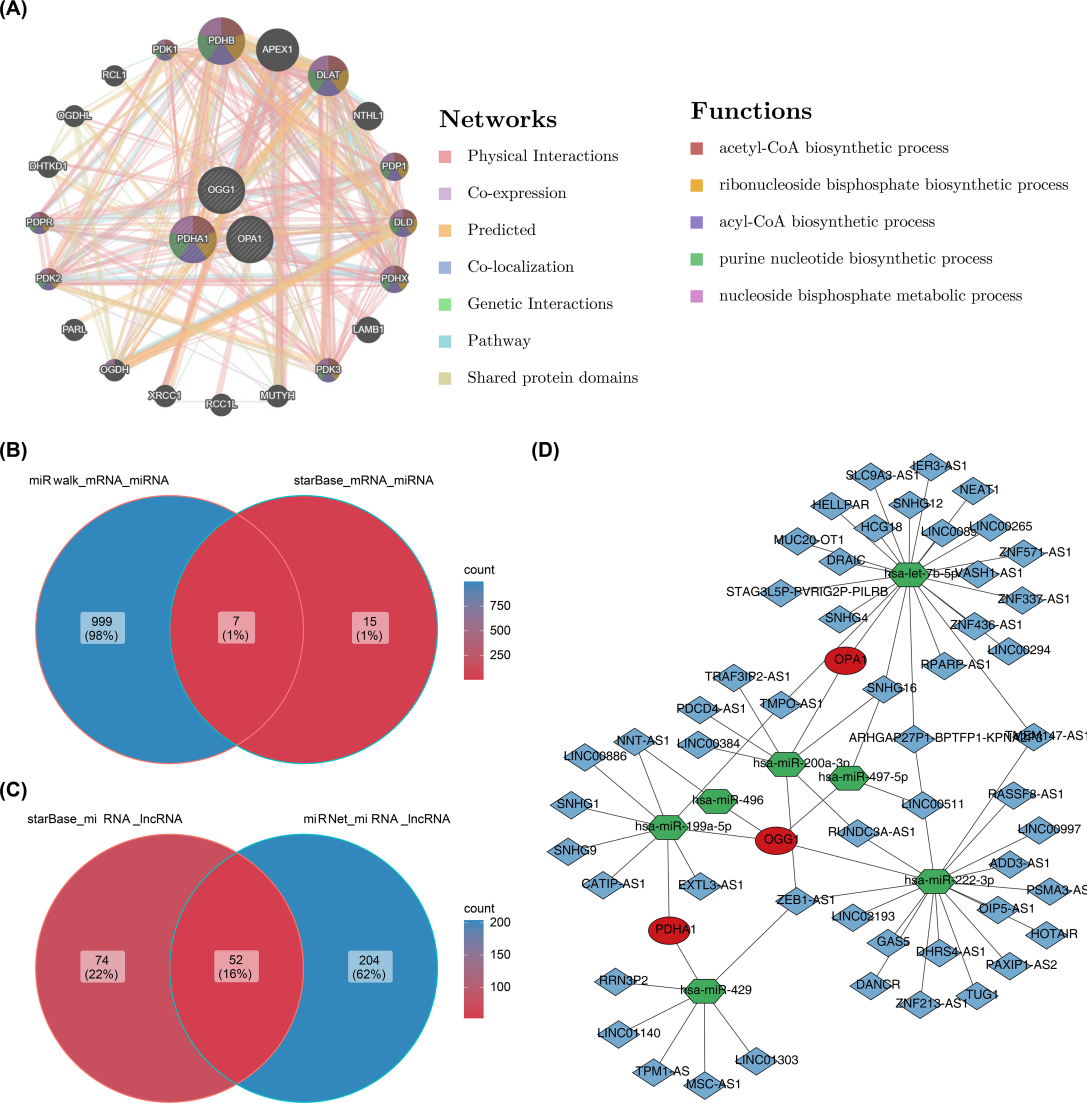
GGI network and molecular regulatory network. **(A)** GGI network of prognostic genes. **(B, C)** Venn diagrams of miRNAs and lncRNAs. **(D)** Regulatory network of mRNA-miRNA-lncRNA interactions.

In the GGI network constructed using prognostic genes and their neighboring genes, involvement in the acetyl-CoA biosynthetic process was identified (**Figure 8A**). The intersection of miRNAs and lncRNAs predicted by different databases resulted in 7 miRNAs and 52 lncRNAs (**Figures 8B, C**). These were used to construct an mRNA-miRNA-lncRNA regulatory network, revealing that hsa-miR-199a-5p regulated both PDHA1 and OGG1 (**Figure 8D**). However, this regulatory relationship remains a hypothetical deduction and requires experimental verification for confirmation.

### 3.8 Prediction of drugs and small-molecule inhibitors

**Figure 9:**
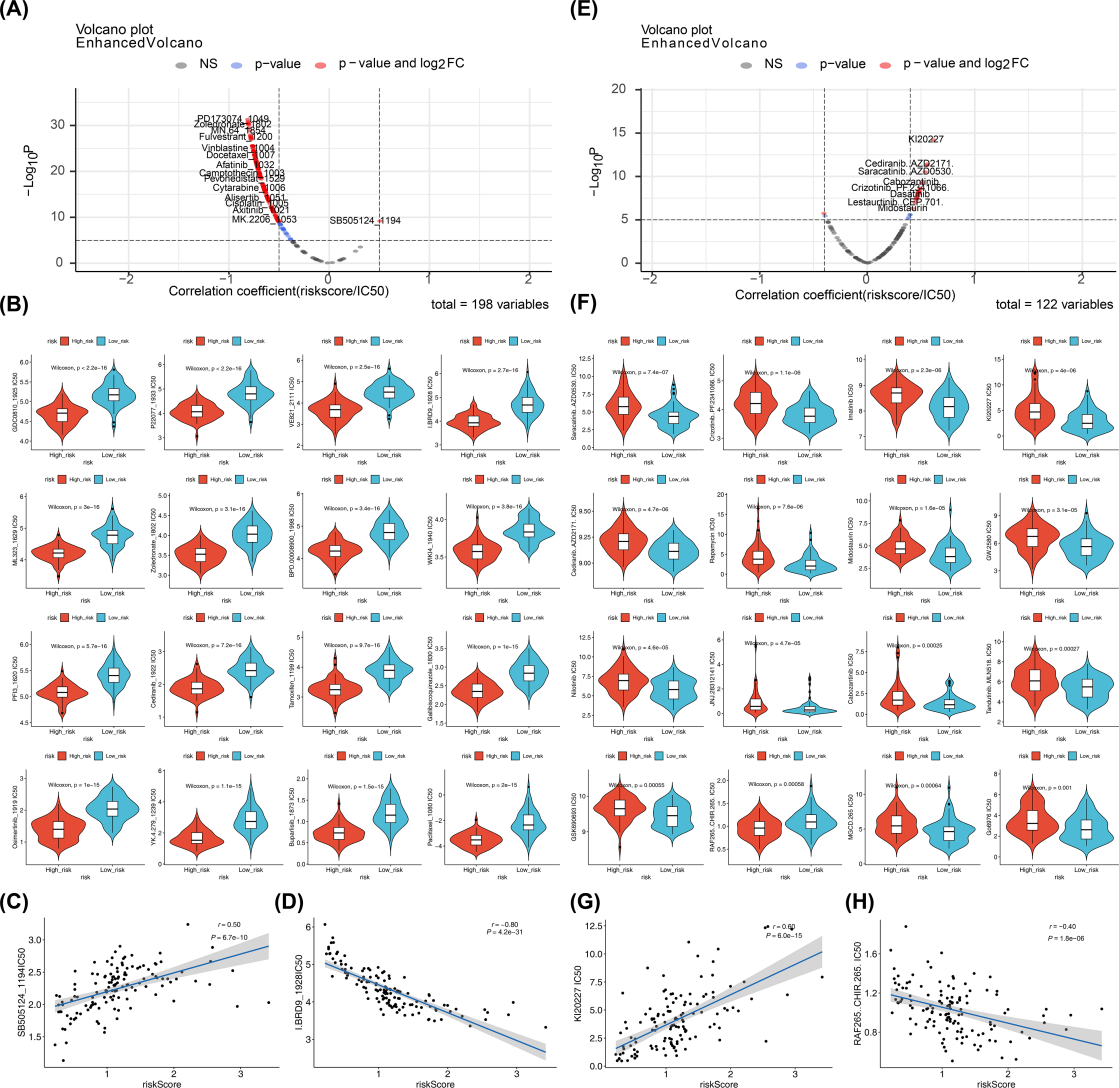
Drugs and small-molecule inhibitors prediction. **(A, E)** Correlations between predicted drug sensitivity (IC50) and risk scores in high- and low-risk groups. IC50 values for chemotherapies/targeted therapies were predicted using oncoPredict based on GDSC; correlations were tested by Spearman (or Pearson if specified) with reported correlation coefficient (r) and p value; thresholds for reporting: |cor| > 0.5 and p < 0.05 for GDSC drugs, |cor| > 0.4 and p < 0.05 for Beat AML small molecules. **(B, F)** Boxplots of predicted IC50 values between high- and low-risk groups (Wilcoxon rank-sum test; two-sided; p < 0.05 considered significant). **(C, D, G, H)** Scatterplots showing correlations between selected compounds (SB5051241194, I.BRD91928, KI20227, RAF265 CHIR.265) and risk scores; correlation coefficients and p values shown; ns (p > 0.05).

Screening 198 drugs from the GDSC database and 122 small-molecule inhibitors from the Beat AML dataset identified 145 drugs and 22 small-molecule inhibitors with significant differences between the risk groups in the training set, including osimertinib, buparlisib, saracatinib, and crizotinib (**Figures 9A, B, E, F**; **Supplementary Tables 13, 14**). In both the GDSC and Beat AML datasets, SB505124_1194 (cor = 0.50, p < 0.0001) and KI20227 (cor = 0.60, p < 0.0001) were the drugs and small-molecule inhibitors most positively correlated with the risk score, while I.BRD9_1928 (cor = -0.80, p < 0.0001) and RAF265 CHIR.265 (cor = -0.40, p < 0.0001) were the most negatively correlated (**Figures 9C, D, G, H**). These candidate compounds are still in the exploratory phase, and their efficacy in pediatric AML must be validated through *in vitro* and *in vivo* studies before clinical translation can be considered.

### 3.9 Validation of prognostic gene expression

**Figure 10:**
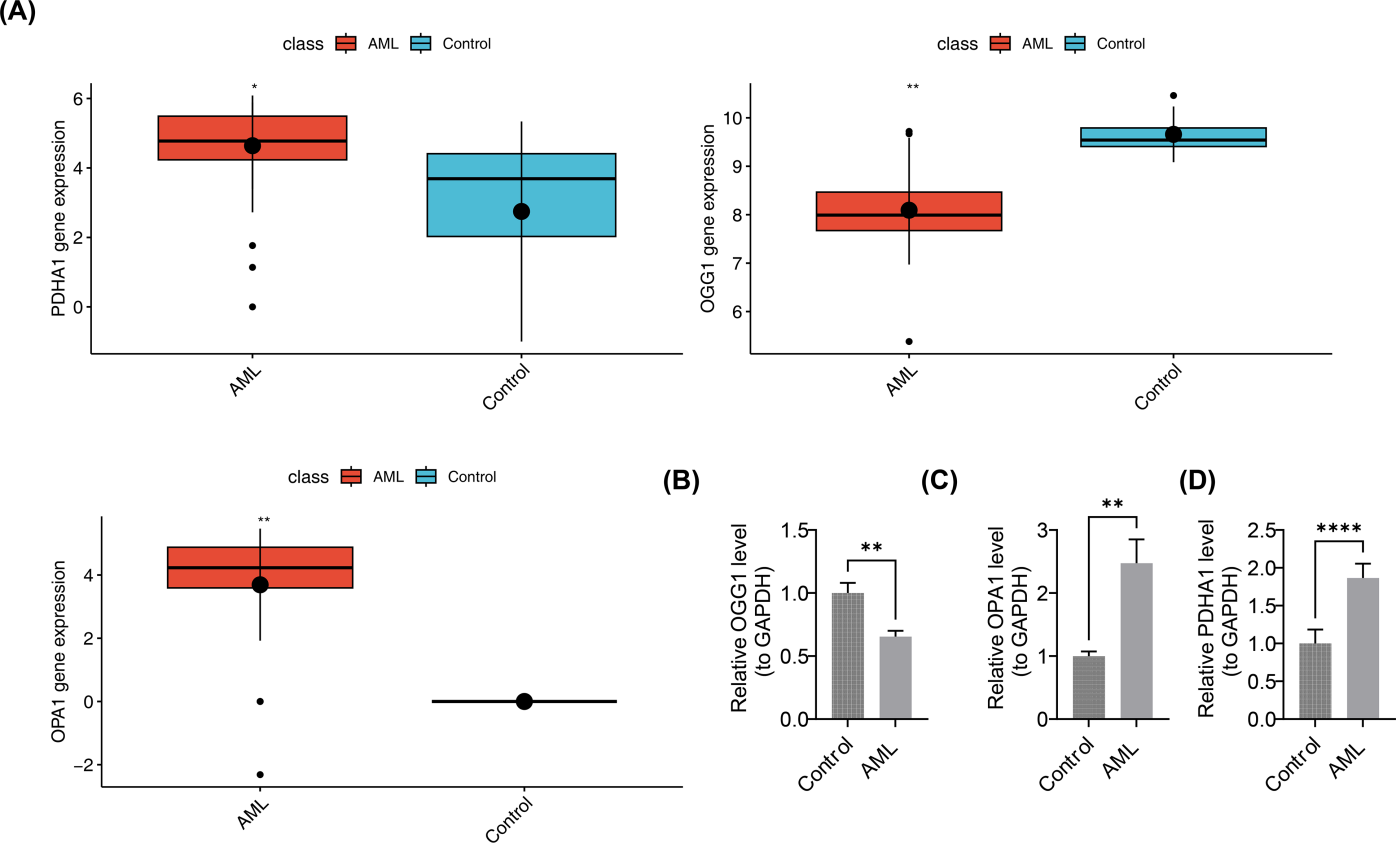
Validation of prognostic gene expression. **(A)** Differential expression analysis of prognostic genes. Boxplots show median and interquartile range; group comparisons by Wilcoxon rank-sum test. **(B–D)** RT-qPCR expression levels of OGG1, PDHA1, and OPA1. n = 5. Expression quantification used 2^^-ΔΔCt^ normalized to GAPDH. Group comparisons used two-sided unpaired Student’s t-test; p < 0.05 considered significant. Significance annotations: ns (p > 0.05); **p < 0.01; ****p < 0.0001.

In the pediatric AML group of GSE2191, PDHA1 and OPA1 were overexpressed, while OGG1 was downregulated (**Figure 10A**). This was validated by RT-qPCR, which showed significant differences in the expression levels of OGG1, PDHA1, and OPA1 between case and control samples (p < 0.05). PDHA1 and OPA1 exhibited higher expression in AML samples compared to controls (PDHA1: p < 0.0001; OPA1: p < 0.01), while OGG1 was downregulated in the AML group (p < 0.01) (**Figures 10B–D**). These results confirmed the consistency between the RT-qPCR findings and the bioinformatics analysis.

## 4 Discussion

As a recently emerging disease with an unclear etiology, pediatric AML has seen limited progress in primary treatment approaches (35). The gene expression profiles of PCD-RGs and mito-RGs in pediatric AML remain inadequately characterized (36, 37). In this study, three potential prognostic genes—PDHA1, OGG1, and OPA1—linked to PCD and mitochondrial function in pediatric AML were precisely identified through differential expression analysis and machine learning techniques. The prognostic model established demonstrated that high-risk patients exhibited significantly reduced survival rates, a finding independently validated in the GSE192638 dataset, confirming the model’s robustness and generalizability. Furthermore, the nomogram incorporating risk score and WT1 mutation exhibited good predictive power for patient outcomes in pediatric AML (AUC values > 0.6). Functional enrichment analysis revealed critical pathways involved in pediatric AML progression, such as translation initiation, DNA repair, apical surface regulation, and EMT, offering valuable insights into the molecular mechanisms of the disease. Drug prediction identified buparlisib and crizotinib as potential therapeutic agents for pediatric AML, while SB505124_1194 and RAF265 CHIR.265 emerged as promising candidates in the Beat AML dataset. These findings not only deepen our understanding of pediatric AML pathogenesis but also provide actionable targets for drug development.

Initially recognized as a key gene in cuproptosis, PDHA1 plays a pivotal role in the reprogramming of glucose metabolism in tumor cells. It is involved in mitochondrial signaling pathways such as oxidative phosphorylation, cellular respiration, and electron transfer activity (38). In AML, PDHA1 mRNA expression is typically reduced, whereas its expression is notably elevated in lymphoid neoplasms, including diffuse large B-cell lymphoma (DLBC) and thymoma (THYM) (38). A 2021 study by Cevatemre et al. showed that silencing PDHA1 expression triggered the EMT in A549 lung cancer cells (39), while Ma et al. found that dichloroacetate activated PDHA1, exerting therapeutic effects in A549 cells, highlighting the critical role of PDHA1 in modulating cellular responses to therapy and tumor progression in non-small cell lung cancer (40). These findings suggest that PDHA1 may have context-dependent roles across different diseases. Notably, while PDHA1 is downregulated in adult AML, our study for the first time reveals elevated PDHA1 expression in pediatric AML, where it correlates with poor prognosis. This observation aligns with previous studies and establishes PDHA1 as a potential diagnostic and prognostic biomarker for pediatric AML, likely through its regulation of mitochondrial metabolism in leukemic blasts. These results not only enhance early diagnostic capabilities but also underscore PDHA1 as a potential therapeutic target for metabolic reprogramming in pediatric AML (38, 41).

OGG1, a well-known DNA repair enzyme, plays a critical role in inflammation modulation and metabolic homeostasis (42). In mammalian cells, OGG1 primarily mediates the removal of 8-oxoG through the base excision repair (BER) pathway. Unrepaired 8-oxoG can lead to G:C to T:A substitution mutations during DNA replication, serving as a biomarker for oxidative DNA damage. Additionally, OGG1 is involved in the transcriptional regulation of nuclear factor kappa B, activation of small GTPases, and inhibition of poly (ADP-ribose) polymerase (PARP)-mediated cell death, all of which are pivotal in modulating inflammation, tumor progression, and age-related disorders (43). In studies of the DNA BER pathway, the OGG1 Ser326Cys polymorphism has been linked to the risk of pediatric ALL: the OGG1 Cys/Cys genotype increases ALL risk, while combined XRCC1/OGG1 or OGG1/MUTYH genotypes confer protection against this malignancy (44). Recent studies in relapsed AML show that low OGG1 expression in leukemic cells correlates with higher mutation burdens (45). However, the prognostic value of OGG1 in pediatric AML remains largely unexplored. Our bioinformatics and qPCR results revealed significantly lower OGG1 expression in pediatric AML, consistent with the findings of Gotoh et al. (45). This suggests that OGG1 is a valuable prognostic marker and offers new insights into biomarker discovery for pediatric AML. However, its direct association with PCD remains unclear and warrants further investigation.

OPA1, a mitochondrial inner membrane GTPase, regulates mitochondrial dynamics, bioenergetics, cristae architecture, and mtDNA stability (46). AML cells are highly reliant on oxidative phosphorylation and mitochondrial dynamics, processes regulated by fusion genes such as OPA1 (47). A recent study showed that pharmacological inhibition of OPA1 with MYLS22 or genetic depletion of mitochondrial fusion genes exerted robust anti-leukemic effects in AML (48). Dysregulated mitochondrial dynamics, including OPA1-mediated fusion, are observed not only in AML but also in other leukemias. A 2019 study by Silic-Benussi et al. demonstrated that the ROS-OMA1-OPA1 axis plays a significant role in drug resistance in pediatric T-cell ALL. ROS scavengers and siRNA-mediated knockdown of the mitochondrial protease OMA1 inhibited OPA1 cleavage and cell death, providing evidence for ROS-targeted therapies in refractory pediatric T-ALL (49). In the present study, high expression of OPA1 was significantly associated with poor prognosis in pediatric AML, confirming its role as a reliable prognostic marker for AML.

In clinical practice, prognostic models are essential for estimating and quantifying patient outcomes (50). The nomogram addresses a critical need in modern medicine by offering a tool to tailor medical decisions to individual risk profiles, aligning with the principles of personalized medicine (51). Compared to existing models, the 1-year AUC for Yang et al.’s pediatric AML stem cell transplantation model was 0.70, while our model achieved an AUC of 0.73 (52). Similarly, Song et al.’s nomogram exhibited a 1-year AUC of 0.62, compared to 0.69 in our study (53). These results highlight the superior accuracy and clinical utility of the prognostic model and nomogram developed in this study, demonstrating their effectiveness in predicting pediatric AML prognosis and providing robust risk stratification and treatment guidance for clinical practice.

During organismal growth and development, the transmission of genetic information and the regulation of cellular functions are essential for maintaining normal physiology (54, 55). Dysregulation of this information, such as abnormal RNA splicing, is a key factor underlying hematopoietic dysfunction in pediatric AML. Recent studies have shown that aberrant splicing triggers DNA damage and impairs repair mechanisms in the pediatric AML hematopoietic system (56). While RAD51 and XRCC3 polymorphisms have been linked to an increased susceptibility to adult AML, combined variant alleles of these DNA repair genes significantly elevate the risk of AML in pediatric populations (57). EMT is a dynamic process involved in embryonic development, inflammation, wound repair, fibrosis, and cancer progression (58). Higher expression of EMT transcription factors, such as ZEB1, correlates with AML progression (58). In an MLL-AF9 oncogene-driven AML mouse model, short hairpin RNA (shRNA)-mediated Zeb1 knockdown reduced bone marrow infiltration *in vivo*, and *in vitro* studies showed impaired tumor cell invasion (59). These findings underscore the role of DNA repair dysregulation and EMT in the spread of leukemic cells, offering critical insights into the pathogenesis of pediatric AML and the development of effective treatment strategies.

Buparlisib, an oral pan-class I PI3K inhibitor, suppresses the PI3K pathway to induce antiproliferative and proapoptotic effects in various tumor types, including ovarian, glioblastoma, breast, and prostate cancers (60). In patients with AML, a daily dose of 80mg buparlisib inhibited the PI3K/AKT/mTOR pathway with acceptable tolerability and preliminary activity (61). Given its broad anti-neoplastic effects, buparlisib has also been tested in AML and ALL, with studies confirming its ability to inhibit PI3K activity, making it a promising treatment for patients with ALL (62). Crizotinib, first approved in 2011, specifically targets anaplastic lymphoma kinase (ALK) (63). Earlier studies have demonstrated crizotinib’s potential in treating hematological cancers with ALK rearrangements. Maesako and Yanagimachi et al. showed that crizotinib effectively reduced leukemia cell burden in patients with ALK-rearranged AML and pediatric AML harboring the RAN-binding protein 2-anaplastic lymphoma kinase fusion gene (64, 65). Together, these agents exhibit significant therapeutic potential for both hematological malignancies, such as AML and ALL, as well as solid tumors. They are expected to offer novel approaches for pediatric AML treatment. However, further research is necessary to fully clarify their efficacy and safety, enabling more optimized clinical applications.

Pediatric AML demonstrated notable sensitivity to two small-molecule inhibitors, SB505124_1194 (a selective TGFβR inhibitor) and RAF265 CHIR.265 (a BRAF inhibitor). These compounds have shown efficacy in various diseases. Given the role of TGFβ signaling in leukemogenesis, SB505124_1194 was tested in pediatric AML. In 2022, Yu et al. reported that pediatric patients with AML could potentially benefit clinically from SB505124_1194 treatment (66). While its efficacy in hepatocellular carcinoma (HCC) is attributed to modulation of the TGFβ pathway (67), this mechanism is also involved in AML stem cell maintenance, supporting its potential for translation into leukemia treatment. Regarding RAF265 CHIR.265, early studies by Khazak et al. demonstrated that RAF265 effectively suppresses wild-type Raf kinases and inhibits mitogen-activated protein kinase (MAPK) signaling in cancer cell lines (68). Preclinical studies in medullary thyroid cancer (MTC) showed synergistic antitumor effects when combined with ZSTK474 (69). In 2023, Li et al. identified a novel application for RAF265 as an antiviral therapeutic against Herpes simplex virus-1 (HSV-1), where its mechanism of action involves regulation of cytoskeleton rearrangement and modulation of cellular translation machinery, highlighting its potential for multitargeted therapeutic applications (68). Together, these small-molecule inhibitors represent valuable candidates for further investigation in pediatric AML. Future research should aim to fully elucidate their mechanisms of action, optimize their therapeutic potential, and explore the possibility of combination therapies with other drugs or treatment modalities to improve outcomes for pediatric patients with AML.

## 5 Conclusions

In this study, transcriptome data and bioinformatics approaches were utilized to identify PDHA1, OGG1, and OPA1 as potential prognostic genes in pediatric AML. The constructed prognostic model and nomogram demonstrate preliminary predictive value, but require further validation in a multicenter cohort. Enrichment analysis linked these genes to genetic information transmission and cellular function regulation pathways between high- and low-risk groups. Through drug prediction analysis, buparlisib, crizotinib, SB505124_1194, and RAF265 CHIR.265 were identified as promising novel therapeutic agents for pediatric AML. Although this approach has provided valuable insights into the pathogenesis and prognosis of pediatric AML, it is not without limitations. Issues related to data quality, inherent assumptions in the employed algorithms, and reliance on RT-qPCR techniques raise concerns. Therefore, additional clinical cohort validation is essential to ensure the robustness and reliability of this method. Concurrently, increasing the sample size and conducting more functional experiments to validate the currently identified prognostic genes are necessary to ensure the research findings stand up to scrutiny in practical applications. Furthermore, with a training sample size of only 131, there is a risk of overfitting. To enhance the generalizability and stability of the results, future studies should explore the possibility of using external cohorts (such as datasets beyond GSE192638) or bootstrapping methods for validation.

Translated with DeepL.com (free version).

## Data Availability

The datasets presented in this study can be found in online repositories. The names of the repository/repositories and accession number(s) can be found in the article/**Supplementary Material**.
