# Supplementary material for: Identification and validation of mitochondrial and programmed cell death-related prognostic markers in pediatric acute myeloid leukemia
Source: Front Immunol. 2025 Nov 20;16:1671230. doi: 10.3389/fimmu.2025.1671230 (PMC12676267; doi:10.3389/fimmu.2025.1671230)
Supplement: Supplementary file 1 [file DataSheet1.zip › Supplementary materials/Supplementary Table 3.docx]

The primer sequences used were as follows:

Supplementary Table 3 RT-q PCR primer sequences

| **Primers** | **Sequences** | |
| --- | --- | --- |
| PDHA1 forward | GACTGTACGCCGAATGGAGT | |
| PDHA1 reverse | AAGGAGAAACTGACCATCACACA | |
| OGG1 forward | GAGGCTGACAGGAGAACAAGA |  |
| OGG1 reverse | TGGGAATCCATCACAGTGCC |  |
| OPA1 forward | GCCACTTCCTGGGTCATTCC |  |
| OPA1 reverse | TTCTGCTATCCAGGCCACAG |  |
| (Internal control)-GAPDH F | ATGGGCAGCCGTTAGGAAAG |  |
| (Internal control)-GAPDH R | AGGAAAAGCATCACCCGGAG |  |
